# Early surgical outcomes and influencing factors of high tibial osteotomy

**DOI:** 10.3389/fsurg.2023.1022636

**Published:** 2023-02-16

**Authors:** Zhaolong Yan, Yange Gu, Jiahuan He, Chenyang Zhang, Jianye Wang, Zhenbin Zhang, Zhang Zhao, Shufeng Li

**Affiliations:** ^1^ Department of Orthopedic Surgery, The First Affiliated Hospital of Shandong First Medical University & Shandong Provincial Qianfoshan Hospital, Shandong Key Laboratory of Rheumatic Disease and Translational Medicine, Jinan, China; ^2^ School of Clinical Medicine, Shandong University, Jinan, China

**Keywords:** unicompartmental osteoarthritis, medial knee osteoarthritis, high tibial osteotomy, surgical effect, influencing factors

## Abstract

**Objective:**

To investigate the influencing factors of functional recovery after high tibial osteotomy (HTO).

**Methods:**

A retrospective research was carried on 98 patients who underwent HTO between January 2018 and December 2020. In each case, the medial proximal tibial angle (MPTA), joint line convergence angle (JLCA), femoral tibial angle (FTA), hip-knee-ankle (HKA), weight bearing line (WBL) ratio of the knee joint, opening gap, opening angle, American knee society knee score (KSS), US Hospital for Special Surgery (HSS) score, Lysholm score, and Western Ontario and McMaster Universities Osteoarthritis Index (WOMAC) were measured to determine postoperative function and influential factors of pain through logistic regression analysis.

**Results:**

The follow-up time was between 18 and 42 months after operation with an average of 27.66 ± 12.9 per month. Overall functional scores were significantly improved. The influencing factors that may affect the postoperative effect of HTO include age and preoperative WBL ratio of the knee joint (WBL%). After incorporating these two factors into the multivariate logistic regression analysis, for every 1 unit increase in the preoperative WBL%, the probability of postoperative HSS being superior is 1.06 times higher than before [Exp(*β*): 1.062, 95% CI: 1.01–1.1, *p* = 0.018]. For every year increase in age, the probability of an excellent HSS score after surgery was 0.84 times higher than that before surgery [Exp(*β*): 0.843, 95% CI: 0.718–0.989, *p* = 0.036]. Preoperative WBL% ≥ 14.37 was 17.4 times more likely to be rated as excellent postoperative HSS than that <14.37 [Exp(*β*): 17.406, 95% CI: 1.621–186.927, *p* = 0.018].

**Conclusion:**

The postoperative functional scores of the patients significantly improved. Patients with preoperative WBL% ≥ 14.37% had better function after surgery.

## Introduction

Osteoarthritis is a joint disease of great concern in the world today and is characterized by synovial hyperplasia and degeneration of articular cartilage with osteophyte formation that can eventually lead to pain, joint loss and disability (1). Degeneration of articular cartilage is most common in the knee joint, mainly in the medial space (2). HTO is based on the concept of force line rearrangement, redistributing weight-bearing and mechanical stress to less disrupted areas and reducing pain, improving function, and achieving articular cartilage recovery (3). This meets the needs of young patients who participate in sports or individuals who want to participate in high-demand activities to avoid prosthesis replacement (4). Historically, HTO was first reported in the 1960s by Jackson et al. (5). In terms of the trend of previous years, an increasing number of literatures related to HTO will be published in the future. HTO combined with cartilage restoration techniques, postoperative prognosis and outcome, and surgical technique research may be the future hotspots in the field of HTO research (6). The main purpose of this study was to re-evaluate the surgical outcome of HTO and the influencing factors affecting the surgical outcome (5, 7–9). This article retrospectively analyzes the clinical data of patients with knee osteoarthritis (KOA) treated with HTO and discusses the factors that affect the postoperative efficacy of HTO.

## Materials and methods

### General information

The research subjects were patients who underwent medial open HTO due to KOA in the First Affiliated Hospital of Shandong First Medical University from January 2018 to December 2020. The inclusion criteria consisted of (a) medial OA of the knee joint with varus deformity mainly in the tibia; (b) age ≤70 years old (higher requirements for future activities); (c) limited extension of the knee joint ≤10° (flexion > 90°); (d) BWI < 40; and (e) no subchondral bone abrasions. The exclusion criteria consisted of (a) patients with a history of lower extremity fracture or surgery on the operative side; (b) traumatic KOA; (c) combined nerve, muscle, or connective tissue diseases; and (d) patients with incomplete imaging parameters and those lost to follow-up.

### Surgical methods

All operations were performed by the same group of physicians, and the anesthesia method used was either intraspinal or general anesthesia. The decision to perform knee arthroscopy is determined according to whether there is a combination of meniscal injury and cartilage injury before surgery. In each case, a 6–8 cm straight incision was made on the medial side of the proximal tibia, exposing the proximal flat joint line layer by layer until the medial tibia and posterior tibial crest were exposed. Two parallel 2.0 cm Kirschner wires were inserted in at 3.5 cm from the upper edge of the tibia and 1.5 cm from the outer edge of the tibia to the upper edge of the tibia to penetrate the contralateral cortex. The horizontal osteotomy was performed close to the distal end of the two Kirschner wires, and approximately 1.0 cm from the lateral cortex was reserved as the hinge point. The starting point of the coronal osteotomy was above the insertion point of the patellar tendon and 110° between the horizontal osteotomy to ensure that the patellar tendon insertion remained attached to the distal side of the osteotomy after the osteotomy was corrected. It was necessary to slowly spread through the bone knife or spreader to achieve the predetermined spread angle in the preoperative plan. The force line was determined by force line rod and x-ray fluoroscopy, and the TomoFix locking plate (Johnson & Johnson) was used for fixation. Different osteotomy gap treatment methods were selected according to the opening angle and bone quality.

### Radiographic measurements and follow-up

**Figure 1:**
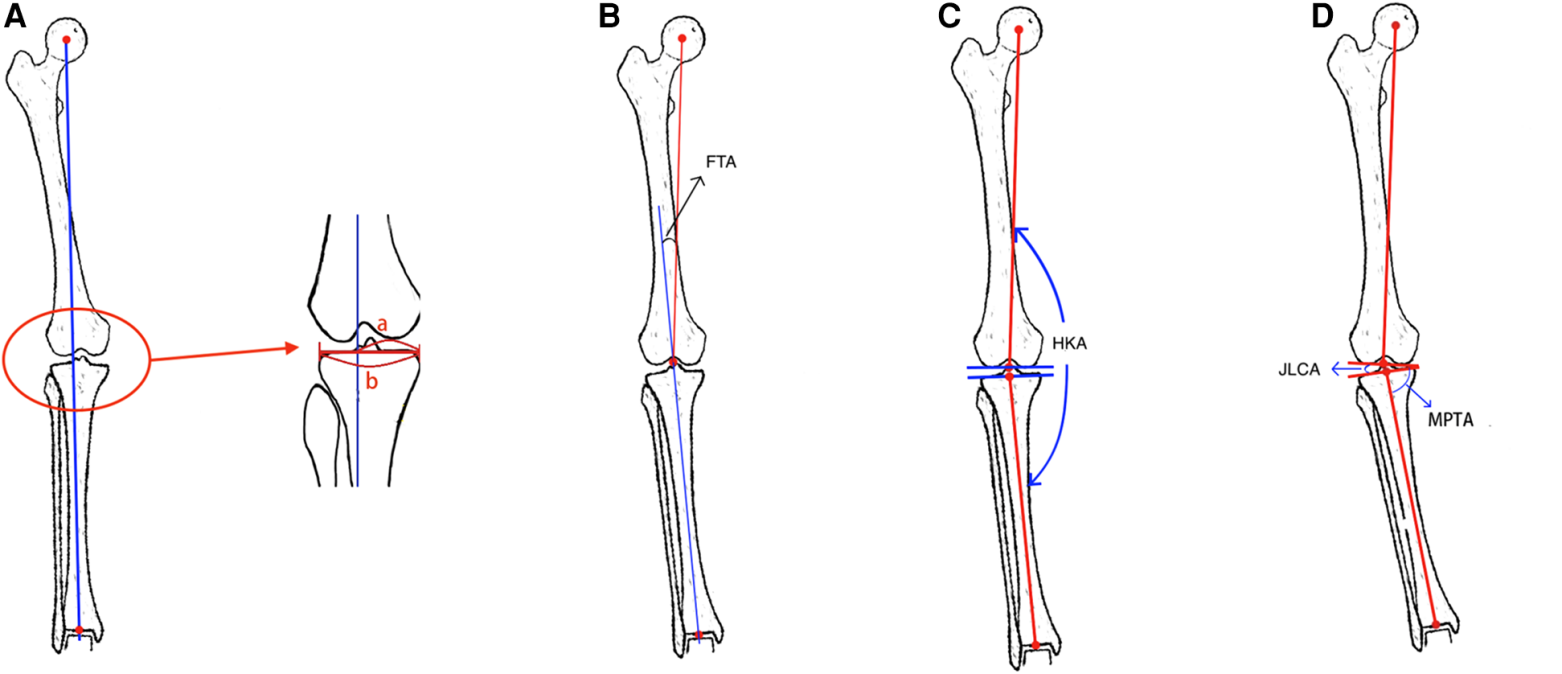
(**A**) Measurement of the WBL: the line connecting the center of the femoral head and the center of the ankle joint is WBL. Measurement of the WBL ratio of the knee joint: a is the distance between the direction of the medial cortex of the tibia along the proximal tibial joint and the force line, b is the distance between the medial and lateral tibial cortex, the ratio of a and b is WBL ratio of the knee joint. (**B**) Measurement of the FTA: the angle between the extension line of the tibial mechanical axis and the femoral mechanical axis is the tibiofemoral angle. (**C**) Measurement of the HKA: the angle between the mechanical axis of the tibia and the mechanical axis of the femor is the hip-knee-ankle angle. (**D**) Measurement of the JLCA: The angle formed between the running direction lines of the opposite joints of the same joint. Measurement of the MPTA: Draw the running direction line of the proximal tibia joint, and draw a straight line from the center point of the ankle joint to the center point of the knee joint on the tibia knee joint line, and the angle between the two lines is MPTA.

**Figure 2:**
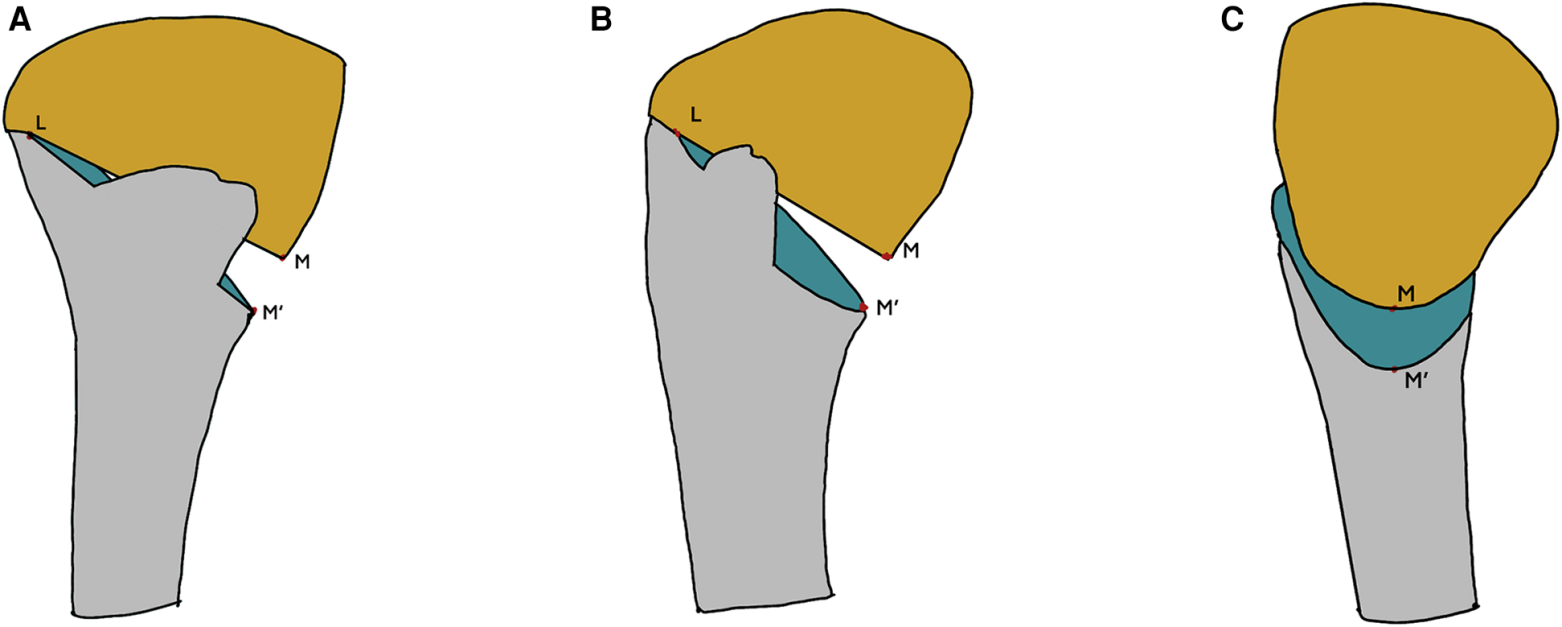
(**A**) Front view. (**B**) 45° projection position view. (**C**) Lateral view. L is the hinge point, M and M’ are the inner cortical points of the upper and lower sections, respectively. Opening gap: the distance between MM’. Opening angle: the angle between LM and LM’.

Radiographic measurements by an independent observer using standard full-length anteroposterior radiographs of both lowed extremities were obtained preoperatively and at least 18 months postoperatively during clinical follow-ups. Imaging parameters included MPTA, JLCA, FTA, HKA, WBL%, postoperative opening gap, and postoperative opening angles (see Figures 1, 2). The postoperative HSS scores were divided into two clinically relevant categories according to the results: excellent ≥85 points and poor <85 points (10).

### Statistical methods

Continuous variables, such as preoperative and postoperative imaging parameters and scores, were tested for normal distribution using the Shapiro-Wilk test. Paired t-tests were performed on normally distributed measurement data, and the results were expressed as mean ± standard deviation ($\bar{X}\pm S$). The paired Wilcoxon signed rank test was used to measure skewed distribution of data, and the paired Wilcoxon signed rank test was used, and the results were expressed as medians (interquartile range) [M (IQR)]. Univariate logistic regression analysis was performed on patients’ general demographic data and various imaging parameters. If the independent variable *p* < 0.05 in the univariate logistic regression appeared in the analysis results, the variable was included in the multiple logistic regression analysis. And differences were considered statistically significant at *p* < 0.05 to determine which variables were the factors affecting the outcome of the functional score and the degree of preoperative pain. All statistical analyses were performed with IBM SPSS Statistics25.

## Results

### Follow-up

During this period, a total of 98 patients received HTO, 10 patients were lost to follow-up, and 2 patients had incomplete imaging parameters. Finally, 86 patients with 94 affected limbs were included in this study. Among them were 8 bilateral cases, all of which were staged operations (41 males and 45 females; average age of 53.38 ± 5.3 years old) (see Table 1).

**Table 1:** General information of the patient.

Parameter	Value
Number of patients (knee)	86 (94)
Age ($\bar{X}\pm S$, year)	53.38 ± 5.3
Height ($\bar{X}\pm S$, meter)	1.64 ± 0.9
Weight [M (IQR), kg]	76 (20)
BMI ($\bar{X}\pm S$, kg/m^2^)	28.53 ± 3.3
**Gender [case (%)]**	
Male	41 (47.7%)
Female	45 (52.3%)
Number of years of disease onset [M (IQR), month]	18 (48)
Follow-up time ($\bar{X}\pm S$, month)	27.66 ± 12.9

### Radiographic improvement

**Figure 3:**
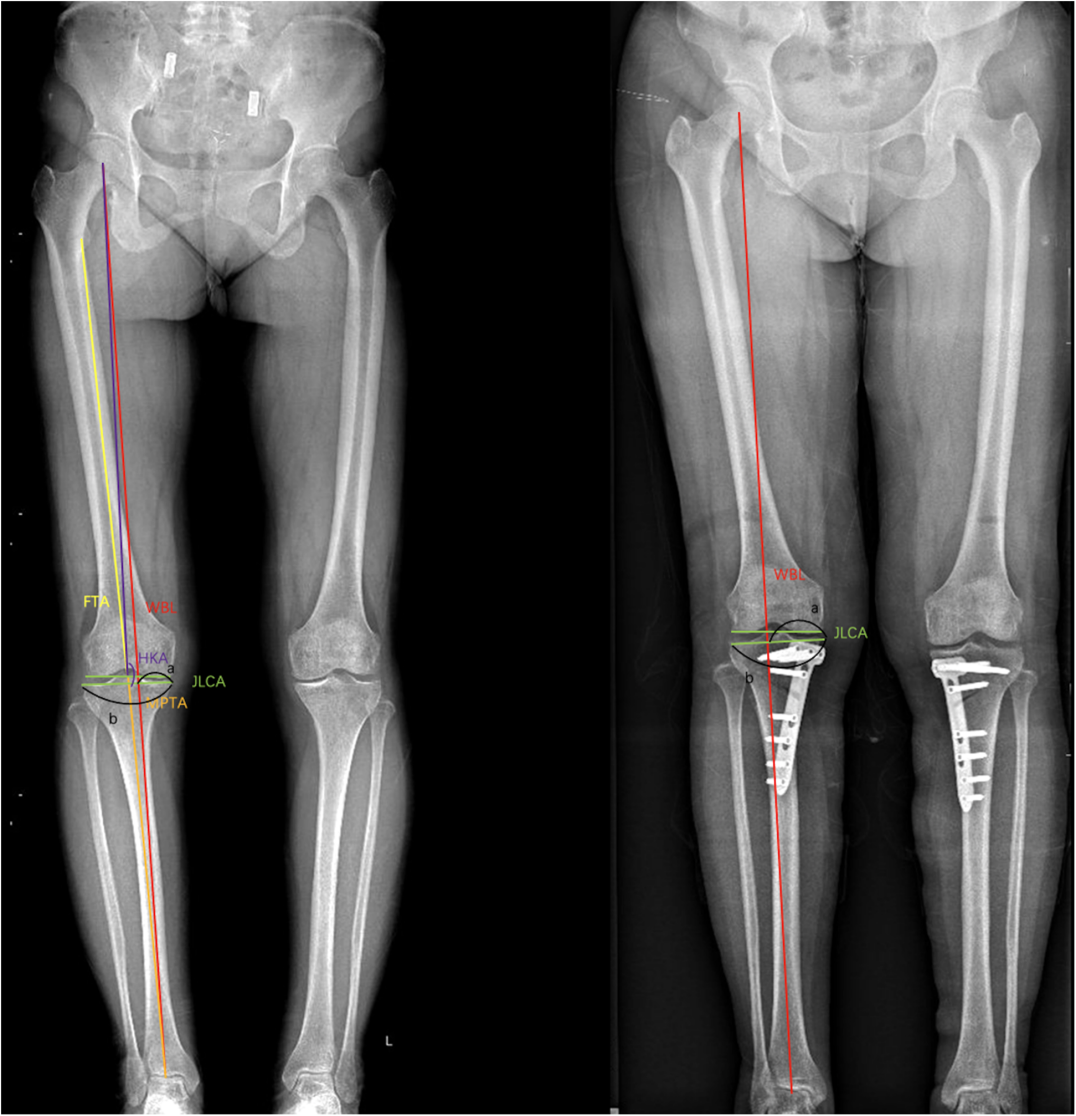
Preoperative and postoperative x-ray comparison. Preoperative WBL ratio of the knee joint: 45%, postoperative WBL ratio of the knee joint: 58%. After operation, the radiographic parameters were significantly improved.

**Figure 4:**
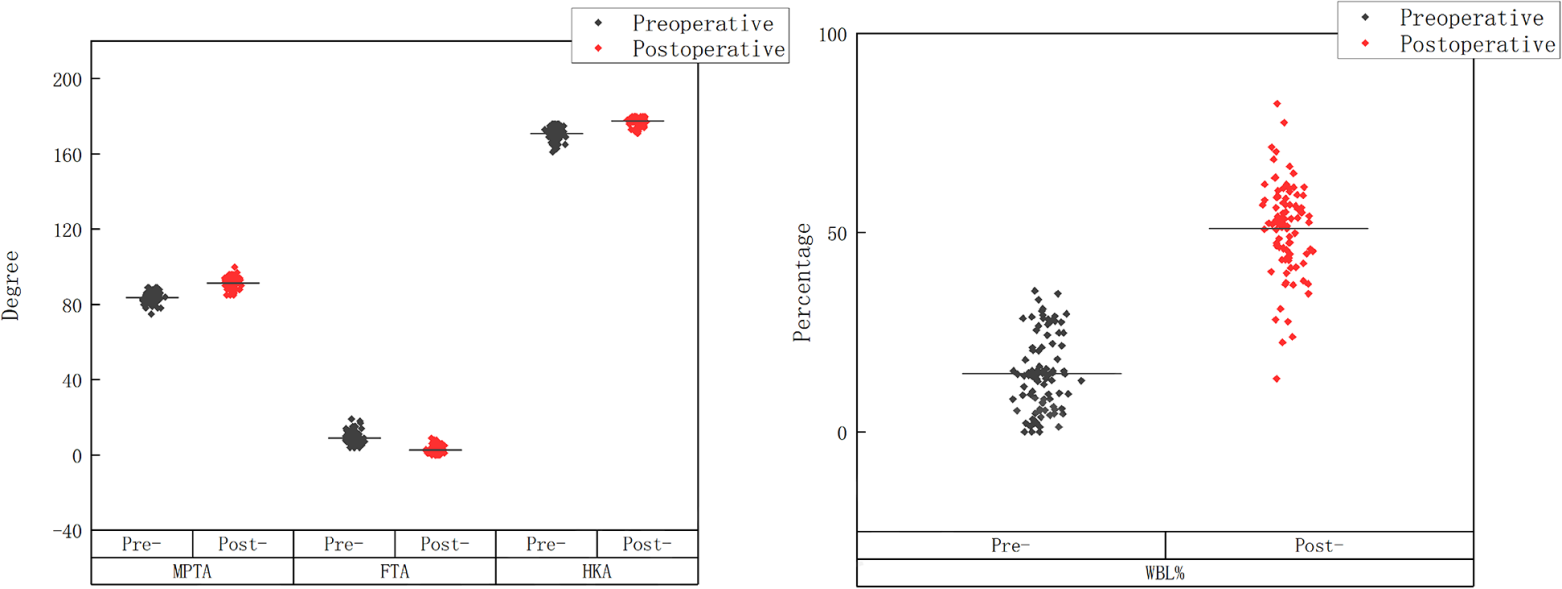
Imaing angle correction trend graph.

The postoperative imaging indicators were significantly improved compared to the preoperative ones (see Table 2, Figures 3, 4). The postoperative MPTA increased, the FTA angle decreased, the HKA angle increased, the WBL% increased, and the differences were statistically significant.

**Table 2:** Preoperative and post-operative x-ray performance of the patient.

Imaging parameters	Preoperative values	Postoperative values	Statistical values	*p* values
MPTA [M (IQR), °]	84 (4)	91 (4)	*Z* = −6.634	<0.001
JLCA [$\bar{X}\pm S$]	3.0 ± 1.6	2.9 ± 1.7	*t* = 1.663	>0.05
FTA [$\bar{X}\pm S$]	8.8 ± 3.4	2.8 ± 2.1	*t* = 12.467	<0.001
HKA [$\bar{X}\pm S$]	174.0 ± 4.8	177.8 ± 2.2	*t* = −7.087	<0.001
WBL ratio of the knee joint [$\bar{X}\pm S$]	17.4% ± 1.4%	50.0% ± 11.4%	*t* = −17.943	<0.001

### Postoperative clinical function comparison

**Figure 5:**
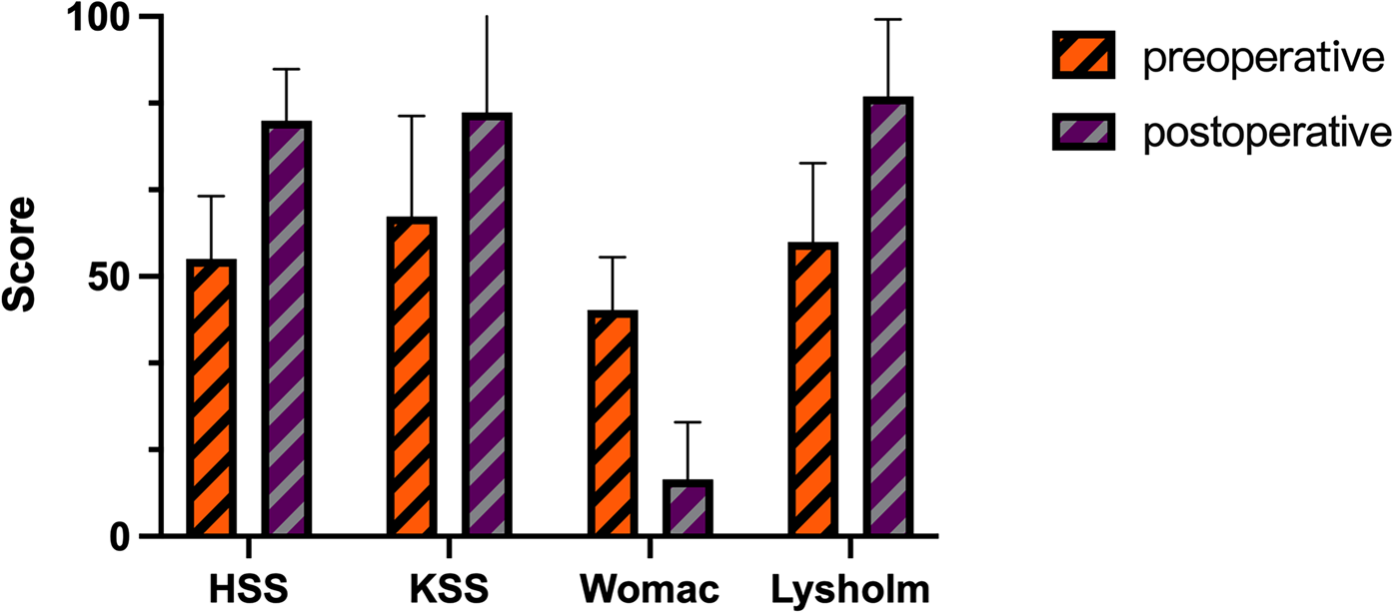
Histogram comparison of HSS, KSS, lysholm and WOMAC. Preoperative womac.

The HSS, KSS, and Lysholm scores significantly increased before and after HTO, the WOMAC score decreased, the difference was statistically significant (see Table 3 and Figure 5), and knee function improved.

**Table 3:** Comparison of preoperative and postoperative clinical outcomes of the knee joint.

Parameters	Preoperative	Postoperative	Statistical values	*p* values
HSS [$\bar{X}\pm S$]	53.4 ± 12.1	77.1 ± 11.0	*t* = −13.466	<0.001
KSS [$\bar{X}\pm S$]	61.6 ± 19.3	75.2 ± 23.9	*t* = −4.051	<0.001
Lysholm [$\bar{X}\pm S$]	56.6 ± 15.1	80.4 ± 18.4	*t* = −8.435	<0.001
Womac [M (IQR), score]	44.0 (9.0)	11.0 (18.0)	*Z* = −7.030	<0.001

### Influencing factors of function after HTO

General information of the patients (age, gender, height, weight, BMI, and age of onset), imaging parameters (MPTA, JLCA, FTA, HKA and WBL%), and whether bone grafting was performed during surgery independent variable screening through univariate logistic regression analysis. Taking the postoperative HSS score category as the following outcome variables, the *p* value was set to 0.05, and the corresponding independent variables were screened out: age (*p* = 0.016) and preoperative WBL% (*p* = 0.031; see Table 4).

**Table 4:** Univariate analysis of postoperative HSS scores.

Variables	*β*	Wald	OR	95% CI	*p*
Gender	−0.154	0.095	0.857	0.322–2.283	0.758
Age	−0.113	4.707	0.893	0.807–0.989	0.030
Height	0.763	0.058	2.144	0.004–1043.41	0.809
Body weight	0.012	0.332	1.012	0.971–1.055	0.564
BMI	0.049	0.331	1.050	0.888–1.242	0.565
Years of disease onset (months)	−0.011	1.107	0.989	0.970–1.009	0.293
With or without bone graft	−0.345	0.083	0.708	0.068–7.352	0.773
Preoperative HSS	−0.022	0.862	1.022	0.976–1.069	0.353
Preoperative Lysholm	−0.003	0.032	0.997	0.962–1.033	0.857
Preoperative Womac	−0.047	2.826	0.954	0.903–1.008	0.093
Preoperative KSS	0.021	1.794	1.021	0.990–1.053	0.180
Preoperative MPTA	0.057	0.235	1.059	0.841–1.334	0.628
Postoperative MPTA	0.073	0.620	1.076	0.896–1.292	0.431
Preoperative JLCA	−0.030	0.024	0.970	0.662–1.423	0.877
Postoperative JLCA	−0.135	0.618	0.874	0.625–1.223	0.432
Preoperative FTA	−0.103	1.036	0.902	0.740–1.100	0.309
Postoperative FTA	0.163	1.673	1.177	0.920–1.505	0.196
Preoperative HKA	0.075	2.113	1.078	0.974–1.194	0.146
Postoperative HKA	−0.099	0.860	0.905	0.734–1.117	0.354
Preoperative WBL%	0.055	4.632	1.057	1.005–1.112	0.031
Postoperative WBL%	0.041	2.718	1.042	0.992–1.094	0.099
Opening gap	0.099	3.706	1.105	0.998–1.222	0.054
Opening distance	0.070	2.772	1.072	0.988–1.164	0.096

The age and preoperative WBL% were included in the multiple logistic regression analysis, and a statistically significant difference was found between age and the preoperative WBL%. According to the multiple logistic regression analysis, each unit increase in the preoperative WBL% was associated with a 1.06-fold increase in the probability of being classified as excellent in the postoperative HSS score [Exp(*β*): 1.062, 95% CI: 1.01–1.1, *p* = 0.018; see Table 5].

**Table 5:** Results of multiple logistic regression analysis of age and preoperative WBL%.

Parameters	*β*	Wald	OR	95% CI	*p*
Age	−0.171	4.391	0.843	0.718–0.989	0.036
Preoperative WBL%	0.060	5.560	1.062	1.010–1.116	0.018

**Figure 6:**
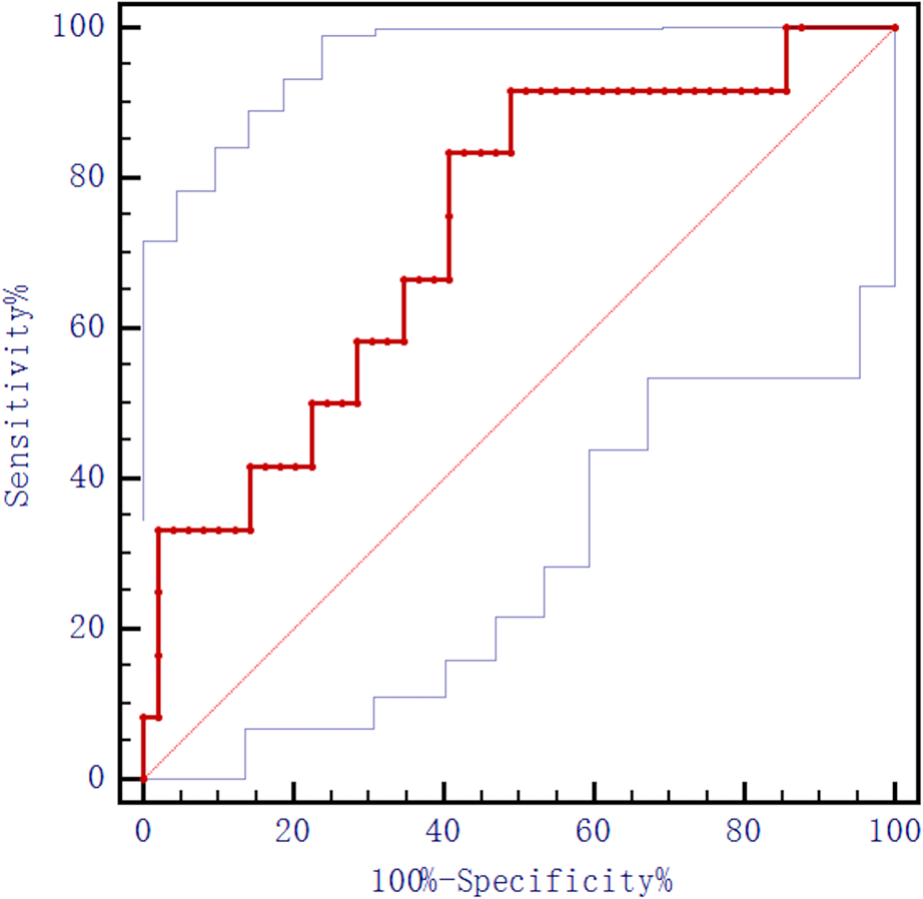
Preoperative WBL ratio of the knee joint ROC analysis.

For each year increase in age, the postoperative HSS score was 0.84 times more likely to be classified as excellent [Exp(*β*): 0.843, 95% CI: 0.718–0.989, *p* = 0.036]. Taking the postoperative HSS score as a state variable and age as a test variable, an ROC analysis was performed on age, and the result found that the area under the curve was 0.323 with no cutoff value found. Taking the postoperative HSS score as the status variable and the preoperative WBL% as the test variable, the ROC analysis of the preoperative WBL ratio in the knee joint showed an area under the curve of 0.731 with a cut-off value of 14.37% (see Figure 6). The preoperative WBL% continuous variables were classified (≥14.37% and <14.37%) and included in the multiple logistic regression analysis. This study has confirmed that the preoperative WBL% ≥ 14.37% had an excellent probability of a postoperative HSS score compared with the preoperative WBL% < 14.37% 17.4 times [Exp(*β*): 17.406, 95% CI: 1.621–186.927, *p* = 0.018; see Table 6] with is a statistically significant difference.

**Table 6:** Results of multiple logistic regression analysis of age and preoperative WBL% ≥ 14.37%/preoperative WBL% < 14.37%.

Parameters	*β*	Wald	OR	95% CI	*p*
Age	2.000	4.561	0.819	0.682–0.984	0.033
Preoperative WBL% ≥ 14.37%/<14.37%	2.857	5.563	17.406	1.621–186.927	0.018

## Conclusion

The present study found significant improvement in functional scores and imaging in patients after HTO, and found a certain correlation between age, preoperative knee WBL ratio and postoperative functional recovery. Although reports on the influencing factors of postoperative function after HTO are gradually increasing, the influencing factors of postoperative function are still inconclusive. Compared with UKA, HTO reduces pain and improves function by changing the position of the force line and improves the conditions for cartilage repair (11). HTO can effectively solve the biomechanical and structural abnormalities in patients with knee osteoarthritis, improve the knee joint function and quality of life of patients, and slow down the progression of degenerative osteoarthritis (12, 13). Because the lateral closed wedge high tibial osteotomy has a higher chance of complications and a higher risk of damage to the common peroneal nerve, the main application is the medial open wedge high tibial high osteotomy (14, 15). Different surgical methods may have an impact on postoperative function, for the convenience of comparison, this paper only collects patients with medial wedge HTO. Several research studies, including the one conducted by Fujisawa et al. (16), found that the adjustment of postoperative alignment of 62.5% is beneficial in improving the effect of HTO. Martay (17) proposed that the WBL% interval should be adjusted to 50%–60% from the inside to the outside because this is more conducive to postoperative recovery. Yoon (11) reported that the postoperative WBL% can be used as a predictor of deterioration of patellofemoral cartilage status. Although there are many reports on the WBL%, the relationship between preoperative knee WBL ratio and postoperative function of patients is lacking. To a certain extent, this study has illustrated the association between the preoperative WBL% and patient function after HTO, meaning that patients with a higher preoperative WBL% have better postoperative function. The ROC curve analysis of preoperative WBL% and the inclusion of multiple logistic regression analysis showed that the critical value of the WBL% was 14.37%. This is statistically significant with the probability of good recovery for preoperative WBL ≥ 14.37% was 17.4% for WBL < 14.37%. This phenomenon may be related to the degree of preoperative deformity and preoperative function. The more severe the medial cartilage wear before operation, the greater the degree of extra-articular deformity, and the greater the correction angle during operation, the greater the functional improvement after operation.

Previous studies have shown that age is one of the influencing factors affecting the function after HTO (18). Attcan (19) conducted follow-ups for patients over and under 50 years of age that underwent HTO and found that the knee joint scores differed significantly with age and that the difference in follow-up mechanical axis between each age group was also statistically significant. This paper draws similar conclusions. The probability of an excellent postoperative effect is 0.82 times higher for each additional year of preoperative age. However, no relevant critical value was obtained in the ROC curve analysis, and this may be related to the small sample size and short follow-up times.

Consistent with other research findings, it was observed that the clinical function scores and postoperative radiographic parameters of patients after HTO were significantly improved compared with those before operation. In addition, we found that patients with higher preoperative WBL ratios of the knee joint will have better joint function after an operation and that older patients will have worse postoperative results. There are certain limitations to the conduct of this research. First, our study was a retrospective case study, which inherently suffers from various sources of bias, including selection bias, measurement and evaluation bias, and loss to follow-up. Another, our sample size is not large enough. Third, this is a short-term follow-up study and does not provide medium- or long-term follow-up results.

## Data Availability

The raw data supporting the conclusions of this article will be made available by the authors, without undue reservation.
